# A new caudipterid from the Lower Cretaceous of China with information on the evolution of the manus of Oviraptorosauria

**DOI:** 10.1038/s41598-019-42547-6

**Published:** 2019-04-25

**Authors:** Rui Qiu, Xiaolin Wang, Qiang Wang, Ning Li, Jialiang Zhang, Yiyun Ma

**Affiliations:** 1 Key Laboratory of Vertebrate Evolution and Human Origins of Chinese Academy of Sciences, Institute of Vertebrate Paleontology and Paleoanthropology, Chinese Academy of Sciences, Beijing, 100044 China; 2 CAS Center for Excellence in Life and Paleoenvironment, Beijing, 100044 China; 3 University of Chinese Academy of Sciences, Beijing, 100049 China; 4 School of Earth Sciences and Resources, China University of Geosciences Beijing, Beijing, 100083 China; 5 College of Life Sciences, Capital Normal University, Beijing, 100048 China

**Keywords:** Palaeontology, Palaeontology, Palaeontology, Palaeontology

## Abstract

Caudipteridae is a basal clade of Oviraptorosauria, all known species from the Early Cretaceous Jehol Biota of northeastern China. They were one of the first feathered dinosaur groups discovered, and possessed avian-like pennaceous remiges and rectrices. Their discovery provided significant information on early oviraptorosaurian evolution and the origins of birds and feathers. Here we describe a new caudipterid species *Xingtianosaurus ganqi* gen. et sp. nov. from the Lower Cretaceous Yixian Formation of Liaoning Province, China. This new taxon differs from other caudipterids by a small pleurocoel close to the dorsal edge of the lateral surface of the dorsal vertebrate centrum, a humerus longer than the scapula, a proportionally long ulna, a relatively small radiale angle, and a relatively short metacarpal I. The phylogenetic results shows *X. ganqi* is an early diverging caudipterid. It exhibits a mosaic morphology, providing new morphological information on early manual evolution of Oviraptorosauria, and giving new light on the evolution of radiale angle among Coelurosauria.

## Introduction

Oviraptorosauria is a group of unusual maniraptoran theropods from North America and East Asia^1^. Since the first oviraptorosaur had been found^2^, all known oviraptorosaurs were found in Late Cretaceous deposits, until Caudipteridae was reported^3^. So far, Caudipteridae is only reported from the Lower Cretaceous Jehol Group of western Liaoning, China, which is famous for feathered dinosaurs^4^. So far, three species have been assigned to this family, including *Caudipteryx zoui*, *Caudipteryx dongi* and *Similicaudipteryx yixianensis*^3,5–8^. Caudipteridae are early diverging oviraptorosaurs with several primitive features such as teeth in the premaxilla, an unfused mandibular symphysis, and a pubic peduncle dorsoventrally longer than the ischiadic peduncle, which shed light on the early evolution of Oviraptorosauria. Many features, like the unfused dentaries, short tail, and specialized two-fingers manus, make caudipterids an unusual taxon among Oviraptorosauria.

The new species reported here, *Xingtianosaurus ganqi* gen. et sp. nov., is from the Lower Cretaceous Yixian Formation of Wangjiagou (Yixian County, western Liaoning, China). This new theropod shows a mosaic of plesiomorphic and apomorphic morphological features on its manus and sternal plates. Its discovery fills the morphological gap between caudipterids and other oviraptorosaurs.

## Result

### Systematic palaeontology

Oviraptorosauria Barsbold, 1976

Caudipteridae Zhou et Wang, 2000

*Xingtianosaurus* gen. nov

Etymology: XingTian, a Chinese deity recorded in Shanhaijing who continued to fight even after his head had been cut off, in reference to the skull-less holotype; saurus, Greek for lizard.

Type species: *Xingtianosaurus ganqi*

Diagnosis: A caudipterid dinosaur distinguished from other caudipterid taxa by the following combination of characters: small pleurocoel close to the dorsal edge of the lateral surface of the dorsal vertebral centrum, humerus longer than the scapula, proportionally long ulna (as long as humerus), relatively small radiale angle (39°, compared to >48° in other oviraptorosaurs with known radiale angle), extremely short metacarpal I (<40% length of the metacarpal II), small ligament pits on the manual phalanges.

*Xingtianosaurus ganqi* sp. nov.

Etymology: Ganqi, the weapon of Xingtian recorded in Shanhaijing.

**Figure 1:**
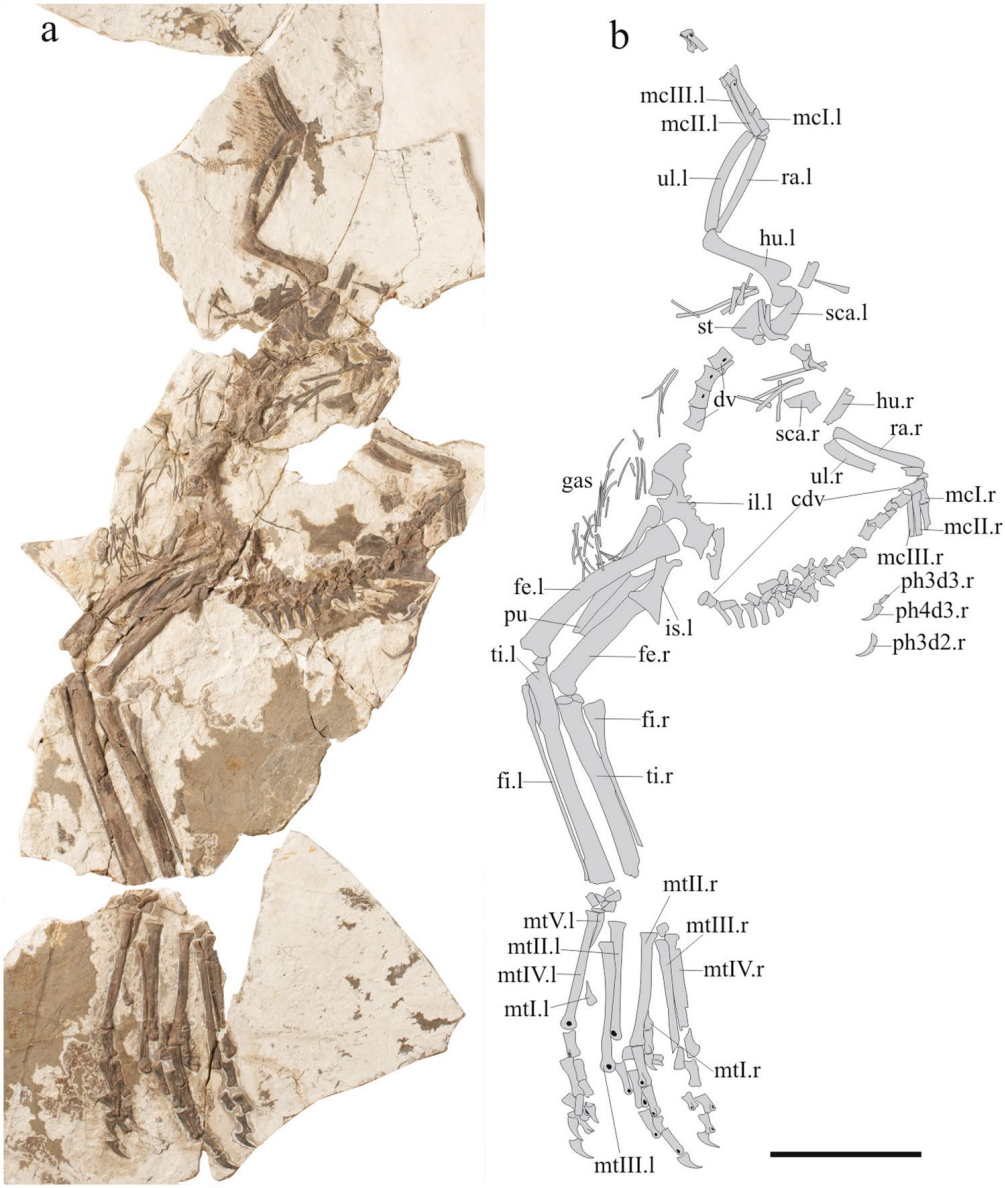
The holotype of *Xingtianosaurus ganqi* gen. et sp. nov. (IVPP V13390). (**a**) Photograph. (**b**) line drawing. Scale bar: 100 mm. cdv, caudal vertebrate; dv, dorsal vertebrate; fe.l left femur; fe.r, right femur; fi.l left fibula; fi.r, right fibula; gas, gastralia; hu.l left humerus; hu.r, right humerus; il.l, left ilium; is.l left ischium; mcI.l left metacarpal I; mcI.r, right metacarpal I; mcII.l left metacarpal II; mcII.r, right metacarpal II; mcIII.l left metacarpal III; mcIII.r, right metacarpal III; mtI.l left metatarsal I; mtI.r, right metatarsal I; mtII.l left metatarsal II; mtII.r, right metatarsal II; mtIII.l left metatarsal III; mtIII.r, right metatarsal III; mtIV.l left metatarsal IV; mtIV.r, right metatarsal IV; mtV.l left metatarsal V; ph3d32.r, right phalanx II-3; ph3d3.r, right phalanx III-3; ph3d4.r, right phalanx III-4; pu, pubis; ra.l left radius; ra.r, right radius; sca.l, left scapula; st, sternum; ti.l left tibia; ti.r, right tibia; ul.l left ulna; ul.r, right ulna. (Photograph by Gao Wei, drawing by R.Q).

Holotype: IVPP (Institute of Vertebrate Paleontology and Paleoanthropology) V13390 (Fig. 1). A partial skeleton, missing the skull, cervical vertebrae, anterior dorsal vertebrae and coracoids.

**Figure 2:**
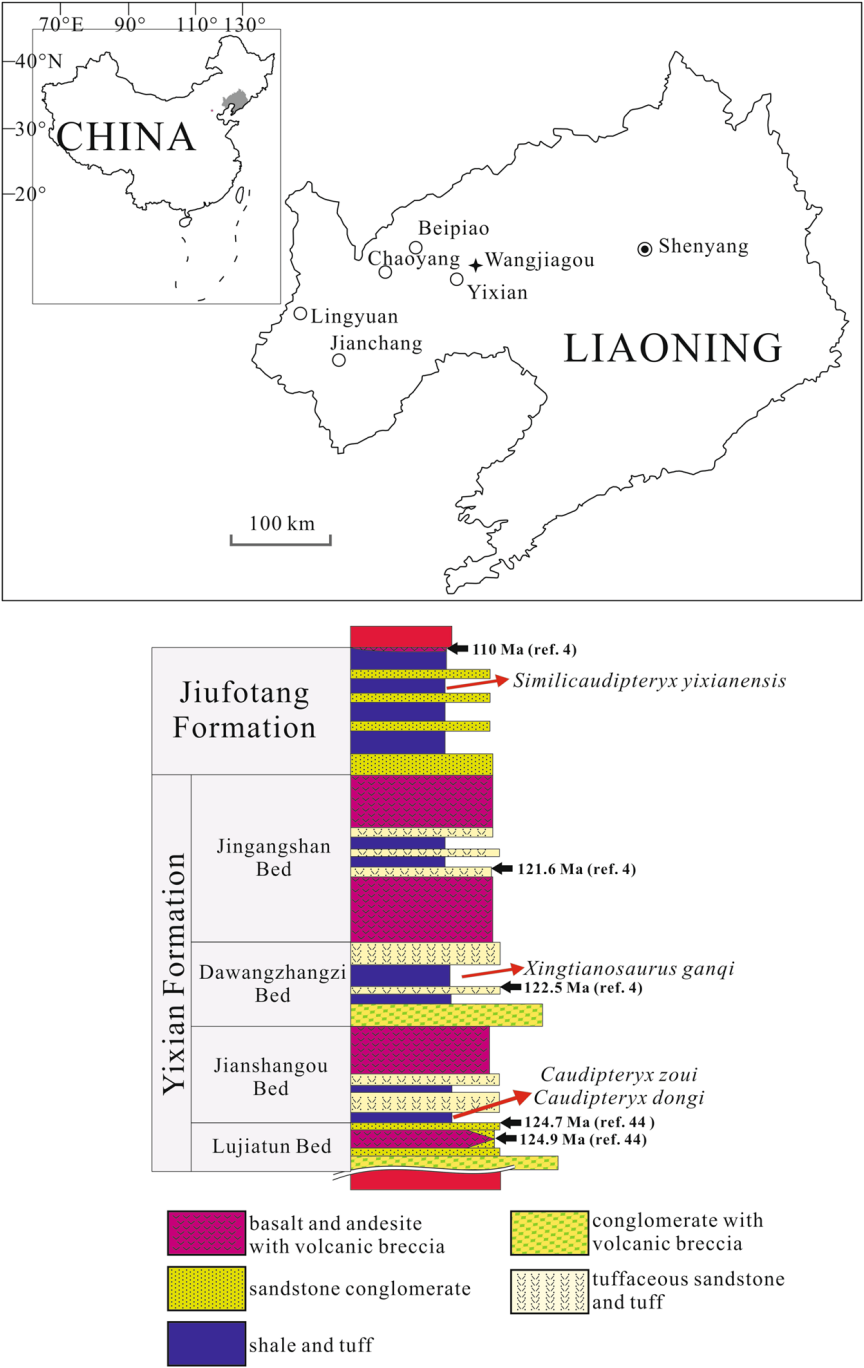
Sketch map and stratigraphic column showing the locality and horizon of *Xingtianosaurus ganqi* gen. et sp. nov. The stratigraphic column is redrawn from Wang & Zhou^4^, with the recently zircon U-Pb ages of Lujiatun and Jianshangou Bed from Yang *et al*.^44^ (Drawing by R.Q).

Locality and horizon: Wangjiagou, Yixian County, Liaoning Province. The Dakangpu Bed (same horizon to Dawangzhangzi Bed) of Yixian Formation, Lower Cretaceous (Fig. 2).

Diagnosis: As for the genus.

### Description

**Figure 3:**
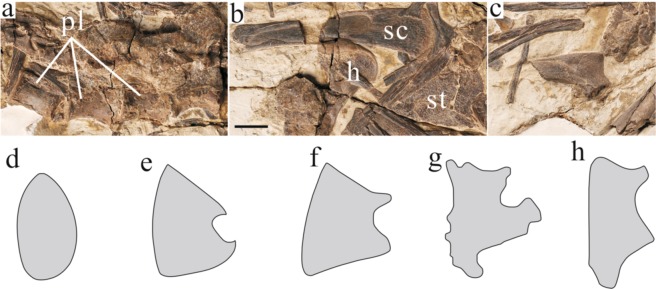
Some postcranial skeleton of *Xingtianosaurus ganqi* (IVPP V13390). (**a**) dorsal vertebrae; (**b**) sternum and left scapula; (**c**) right scapula. Scale bar: 10 mm. (**d–h**) Comparison of sternum among oviraptorosaurs. (not scaled): (**d**) *Caudipteryx*; (**e**) *Xingtianosaurus*; (**f**) *Khaan*; (**g**) *Citipati*; (**h**) *Apatoraptor*. Abbreviations: h, humerus; pl, pleurocoel; sc, scapula; st, sternum. (Photograph by Gao Wei, drawing by R.Q).

Only three centra of the dorsal vertebrae could be observed in lateral view. A small oval pleurocoel is present close to the dorsal edge of the lateral surface of the centrum (Fig. 3a), different from pleurocoel absent in *Caudipteryx* or the deep pleurocoel in *Similicaudipteryx*^7^ and derived oviraptorosaurs^9–11^. The anteroposterior length of the centrum is slightly longer than its height. The ventral margin shows a constriction. Based on the preserved chevrons, at last 18~19 caudals are preserved on *Xingtianosaurus ganqi*. Because the distal most caudals are not preserved, the certain number of caudal vertebrate and whether the distal caudals fused into a pygostyle, as in *Similicaudipteryx*^7^ and *Nomingia*^9^, is unknown. As in other oviraptorosaurs, an obvious transition point is absent^1^. The ratio between the length and height of the vertebrae gradually increases from proximal to distal. However, on the distal most preserved caudals, this ratio is reduced. The long and slender prezygapophyses of middle caudals extend anteriorly, and are longer than the half length of the corresponding centrum. The postzygapophyses are much shorter than the prezygapophyses, and only slightly extend past the posterior articular faces of the centra. The first six chevrons are dorsoventrally elongated and flattened mediolaterally. The middle chevrons are distinctly shortened in height. All chevrons with a preserved distal end show an anterior expansion, which creates a boot-shaped end. As in *Nomingia*^9^, the proximal end of each chevron is divided into three prominent processes. The anterior and posterior processes articulated with the previous and succeeding centrum respectively, and the middle process inserts between the two centra. The dorsal ribs are scattered. The neck separating the capitulum and tuberculum has a concave dorsal margin.

The scapular blade is straight and strap-like (Fig. 3b), unlike the slightly bowed scapula of *Similicaudipteryx*^7^. It is shorter than the humerus, in contrast to other oviraptorosaurs^1^ but similar to paraves^12^. The lateral surface of shaft is strongly concave, making a sharp rim at the dorsal and ventral margins. This concavity extends nearly to the distal end and has a round posterior margin. The dorsal and ventral margins of the scapula are subparallel at mid-shaft and distinctly expanded at the distal end. The distal end is slightly rugose, for the attachment of M. deltoideus scapularis^13^. The acromion is not as prominent as other oviraptorosaurs outside of *Caudipteryx*^1^. It projects anteriorly and slightly dorsally (Fig. 3c).

One sternal plate is preserved but is mostly covered by other bones. It is more than 25% length of femur. This ratio is more similar to oviraptorids such as *Khaan*^11^ and *Citipati*^14^, rather than the small sternum of *C. zoui* (20.69%)^6^ and *C. dongi* (16.45%)^5^. The anterior margin of the sternum is slightly convex for articulation with the coracoid. The sternum bears anterolateral and xiphoid process as Oviraptoridae^11,14^ (Fig. 3e–g) and Caenagnathidae^15^ (Fig. 3h), unlike the two oval sternal plates in *Caudipteryx*^3,5^ (Fig. 3d). The differences between the sternum of *Xingtianosaurus* and *Caudipteryx* are unlikely to be the result of ontogeny for the following reasons: the sternum of *Caudipteryx* is connected with the sternal ribs, with no space for the existence of cartilage^3,5^; and the fusion of the centra and neural arches, the well-ossified wrist and ankle bones, absence of pits and grooves on the surface of bones indicate the adult nature of *Caudipteryx*^3,5^. The sternum of *Xingtianosaurus* has a wrinkled surface for attachment of M. pectoralis^13^.

As in caudipterids, the humerus is half the length of the femur. The humeral head is pronounced and domed at its proximal end. The deltopectoral crest is broken but extends less than one-third of the shaft. The internal tuberosity is located in nearly the same plane as the humeral head and extends a short distance. All that can be said about the distal end is that is slightly expanded and has a nearly round lateral condyle. The ulna is subequal in length to the humerus, which differs from most maniraptors but is shared with some avialae^16^. The olecranon process is weakly developed and smaller than the coronoid processes. The shaft bows posteriorly, which is a synapomorphy of Maniraptora^17^. The distal end shows no dorsoventral expansion, and is almost as wide as the mid-shaft, in contrast to a pointed end in *Khaan*^11^ and *Anzu*^18^. The radius is slightly bowed anteriorly, this condition could also be seen in some derived oviraptorosaurs^11,14^ but is usually absent in Caudipteridae. The shaft expands in width distally from about 40% of the way from the proximal end.

**Figure 4:**
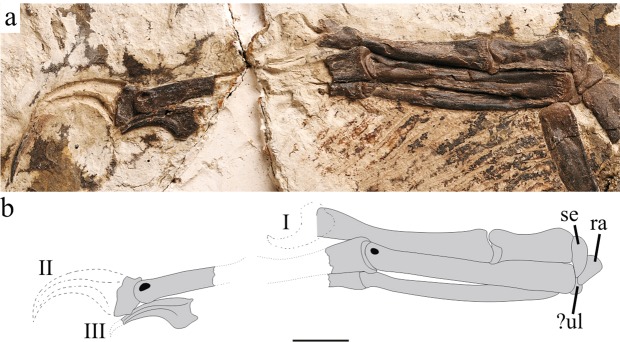
Photograph (**a**) and line drawing (**b**) of manus of *Xingtianosaurus ganqi* (IVPP V13390). Abbreviations: ra, radiale; se, semilunate carpal; ul, ulnare. Scale bar: 10 mm. (Photograph by Gao Wei, drawing by R.Q).

There are three carpals preserved in extensor view (Fig. 4). The radiale is wedge-shaped in dorsal view. It is relatively large, similar in size to the semilunate carpal. The radiale angle^19^ is about 39°. This angle is much smaller than other oviraptorosaurs including *Caudipteryx*, but similar to some deinonychosaurids^19^. There is a small carpal locates between the ulna and radius, articulating with the radiale and metacarpal II, but it is hard to determine whether it is the ulnare or intermedium. As in other oviraptorosaurs^11,15,20^ except *Heyuannia*^21,22^, the proximal surface of the semilunate carpal is not strongly convex as paraves. The distal surface covers the entire proximal end of metacarpal I and half of metacarpal II. Metacarpal I is slightly more robust than metacarpal II and less than 40% of the length of the second (Fig. 4). This ratio is smaller than any known oviraptorosaurs (except *Gigantoraptor*^23^) where it is approximately 50% in derived species and larger than 40% in Caudipteridae. The proximal end has a slightly concave articular surface for contacting with the semilunate carpal, different from the nearly flat surface of other oviraptorosaurs. The shaft of metacarpal I contacts tightly with metacarpal II for almost its whole length, whereas in *Machairasaurus* the distal end of metacarpal I is bended and separated from metacarpal II^20^. The distal articular surface is ginglymoid and slightly rotated, making phalanx I-1 point medially when in flexion. The collateral ligament pits are absent on the distal end. The shaft of metacarpal II is nearly straight and the proximal surface is nearly flat. On the distal end, the collateral ligament pit is relatively small and dorsally located. Metacarpal III is more than 90% length of the metacarpal II. The shaft of metacarpal III is compressed with a groove on the medial surface for contact with metacarpal II. The proximal end is bended medially and does not reach the proximal end of metacarpal II. Phalanx I-1 is the longest phalanx and has a weakly concave proximal articular surface as other caudipterids. In contrast, derived oviraptorosaurs always have a deep concave surface^5,6,11,14,20,24,25^. Phalanx II-1 is preserved, and it also has a weakly concave articular surface. On all phalanges with a preserved distal end, ligament pits are small and dorsally located. This condition is more similar to derived oviraptorosaurs, while Caudipteridae have a large circular ligament pit near the center. Almost all non-ungual phalanges of digit III are taphonomically missing except the penultimate phalanx on right hand. The shaft of this phalanx is very slender with a relatively large and circular ligament pit. All unguals are strongly curved with a prominent flexor tubercle. The proximodorsal lip is absent.

**Figure 5:**
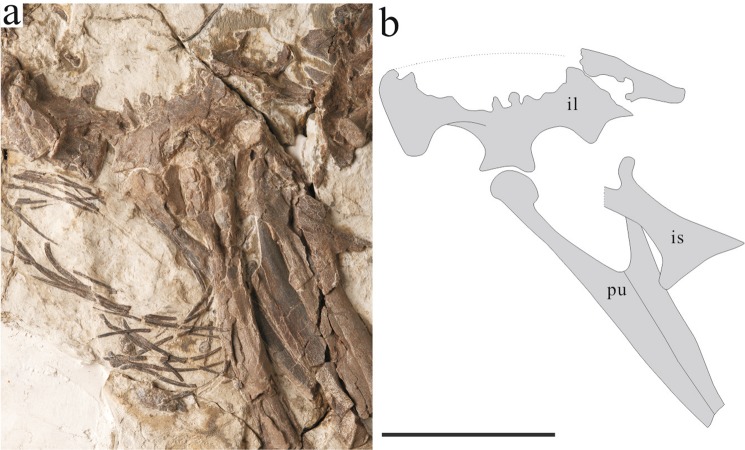
Photograph (**a**) and line drawing (**b**) of pelvic girdle of *Xingtianosaurus ganqi* (IVPP V13390). Abbreviations: il, ilium; is, ischium; pu, pubis. Scale bar: 50 mm. (Photograph by Gao Wei, drawing by R.Q).

The ilium is badly preserved (Fig. 5). The anteroventral process does not extend further than the pubic peduncle, unlike in *Caudipteryx* (IVPP V12430)^6^. The cuppedicus fossa is well-developed, with a somewhat arched ventral margin, unlike the nearly straight margin in *Caudipteryx* (IVPP V12430)^6^ and *Nemegtomaia*^10^ but similar to those of *C. dongi*, *Similicaudipteryx*^7^ and derived oviraptorosaurs. The pubic peduncle slightly bends anteroventrally, indicating a propubic condition. In contrast to most maniraptors except *Similicaudipteryx*^7^, the pubic peduncle is constricted at the base and expands anteroposteriorly at the distal ends. Pubis is approximately 86% length of the femur as in Caudipteridae^5,6^. This ratio is about 90% in Caenagnathidae^26,27^ and nearly 100% in Oviraptoridae^11,28,29^. The ratio between pubic symphysis to pubis is 57% (Fig. 5), similar to *Similicaudipteryx*^7^, but larger than 50% in *Caudipteryx*^5,6^ and *Microvenator*^26^. This ratio in most caenagnathoids with known pubic symphysis is larger than 60%^11,23,28^. The ischium is plate-like. The dorsal margin is strongly bowed anteriorly. As in other oviraptorosaurs, a large triangular obturator process is located at mid-shaft of ischium. The lateral surface of obturator process is concave for the attachment of M. adductor femoris 1^30^. The dorsal margin of the obturator process is thickened.

The femoral shaft is bowed anteriorly in lateral view. The proximal end of the femur is broken and it is difficult to observe any details. There is no fourth trochanter. The tibia is approximately 125% the femoral length. On the posterior surface, the lateral and medial condyle are well-developed and slightly point upwards. Both condyles are large and round, and are separated by a shallow groove. A pronounced fibular crest is preserved on the lateral surface of tibia, about 26% of the way down the shaft of the tibia from the proximal end. This ratio is shorter than *Gigantoraptor* and *Khaan*. The mid-shaft of tibia is compressed lateromedially but the distal end expands to be plate-like. The tibia is not fused with the astragalus. The medial surface of fibula is concave for contact with the lateral condyle of tibia. The tubercle for the insertion of M. iliofibularis is not obvious, but is located a quarter way down the shaft. As in other neotheropods, the concavity turns into a narrow groove along the medial surface of fibula^14,31–33^. The groove expands below the iliofibularis tubercle, offering an attachment for the interosseous membrane between tibia and fibula.

The distal tarsals are not fused with the metatarsals. Metatarsal I is strongly reduced, and located three-quarters of the way down metatarsal II. Metatarsal III is the longest, then II and IV, respectively. The proximal end of metatarsal II expands both anteroposteriorly and transversely. Most of the shaft is straight, but the distal quarter is bent medially. Metatarsal III is strongly compressed along more than half of its length. The proximal end of metatarsal III is slightly anteroposterior expanded. The anterior and posterior margins are subparallel to be strap-like, with no obvious proximal constriction, which is seen in *Chirostenotes*, *Leptorhynchos* or some troodontids^34^. Because the proximal end of metatarsal II and IV are not articulated, whether a true arctometatarsal exists is doubtful. The lateral margin of metatarsal IV is constricted to make a sharp ridge. The metatarsal V extends less than one-third of length of metatarsal III, and its shaft is bowed. Metatarsal II to IV bear large and deep ligament fossae on the distal end. The joints of all non-ungual phalanges bear shallow or no ginglymous articular surface. On the distal end of each phalanx there is a deep ligament fossa on both sides. Each phalanx is longer than the distal one, except the fourth phalanx of digit IV, as an adaptation for terrestrial locomotion^35^. The pedal unguals are only slightly curved. A vascular groove extends from proximal end to the tip near the mid-line on both sides, whereas it is more ventrally locates in *Caudipteryx* (IVPP V12430).

Some imprints and fragments of pennaceous feathers are preserved posterior to the forearm and metacarpals (Fig. 4a). The feathers are perpendicular to the ulna and covered the distal half of the forearm. The attachment of remiges is different from known *Caudipteryx* specimens, which possess pennaceous feathers only on the metacarpals^3,5,6^. Details of the plumage are hard to verify because of the bad preservation.

### Systematics of *Xingtianosaurus* ganqi

**Figure 6:**
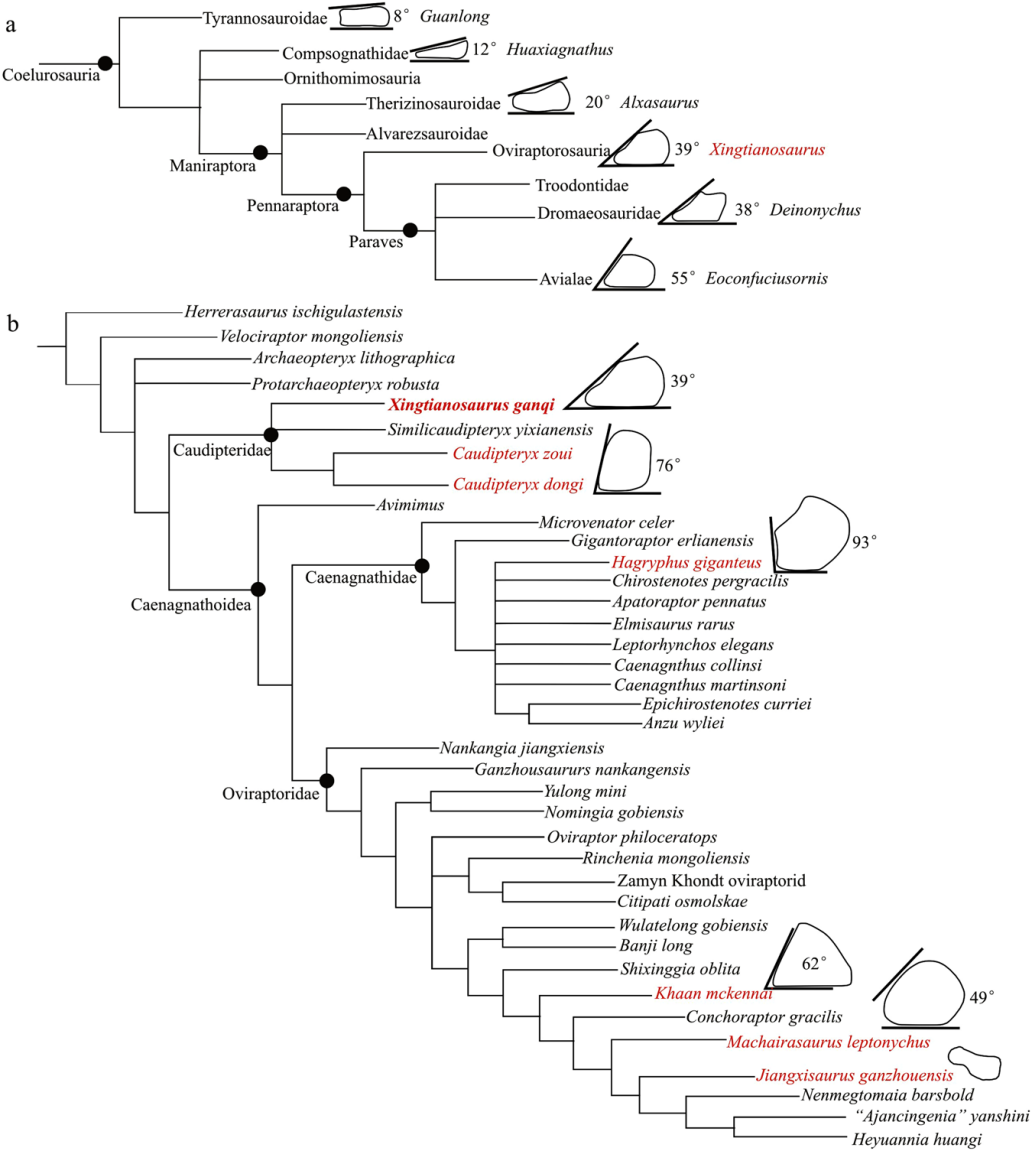
(**a**) Evolution of the radiale angle in coelurosaurian dinosaur. The phylogeny is from the 50% majority rule tree of the first analysis. The radiale angle of *Guanlong*, *Huaxiagnathus*, *Caudipteryx*, *Deinonychus*, *Eoconfuciusornis* is from Sullivan *et al*.^19^; The line drawings of the radiale of *Guanlong*, *Caudipteryx*, *Deinonychus*, *Eoconfuciusornis* are redrawn from Sullivan *et al*.^19^; *Huaxiagnathus* is redrawn from Hwang *et al*.^45^; *Alxasaurus* is redrawn from Russel & Dong^46^. All line drawings are not to scale. (**b**) The strict consensus tree of oviraptorosauria and the evolution of the radiale angle in oviraptorosaurian dinosaur. The line drawings of *Hagryphus* is redrawn from Zanno & Sampson^25^; *Khaan* is redrawn from Balanoff & Norell^11^; *Machairasaurus* is redrawn from Longrich *et al*.^20^; *Jiangxisaurus* is redrawn from Wei *et al*.^42^. All line drawings are not to scale. (Drawing by R.Q).

Because the skull is not preserved, we first investigated the systematic position of *Xingtianosaurus ganqi* by adding it to a coelurosaurian matrix^36^ (Fig. 6a). The analysis produced 74 most parsimonious trees of 3374 steps (Consistency Index = 0.32, Retention Index = 0.776). The result supports its location at the base of Oviraptorosauria, close to *Caudipteryx*. This position is consistent with the primitive morphological features: the tail is proportionally short with fewer than 26 caudals; the humerus is relatively short, about half the length of the femur; metacarpal I is shorter than 50% length of the second; the proximal articular surface of manual phalanx I-1 is slightly concave; and the proximal portion of metatarsal III is compressed transversely.

In order to determine the relationship of *Xingtianosaurus ganqi* gen. et sp. nov. relative to other oviraptorosaurs, we conducted a phylogenetic analysis using an oviraptorosaurian data metrics modified from combination of Osmólska *et al*.^1^, Lamanna *et al*.^18^, and Funston & Currie^15^. The analysis results in 2 most parsimonious trees of 581 steps (Consistency Index = 0.513, Retention Index = 0.691). The strict consensus of the most parsimonious trees confirms that *Xingtianosaurus ganqi* belongs to Caudipteridae (Fig. 6b). In this result, Caudipteridae is only supported by one character: Preacetabular process expanded ventrally well below the level of the dorsal acetabular margin (character 138). However, this analysis gives less information about the relationship between *Caudipteryx*, *Similicaudipteryx* and *Xingtianosaurus ganqi*, which are recovered in a polytomy.

## Discussion

### Manus evolution in oviraptorosauria

*Caudipteryx* can be easily distinguished from other oviraptorosaurs by its bizarre manual characters:^5,6^ a strongly reduced manual digit III with only two short phalanges whose combined length is slightly longer than half the length of phalanx manual II-1; a large ligament pit on the manual phalanges; and an only shallowly concave proximal articular surface of manual phalanx I-1. The hand of *Xingtianosaurus ganqi* provides important information that reduces the morphological gap between *Caudipteryx* and other oviraptorosaurs. As in *Caudipteryx*, the proximal articular surface of phalanx I-1 in *Xingtianosaurus ganqi* is weakly concave, but it shares with derived oviraptorosaurs a relatively small ligament pit. Unlike *Caudipteryx*, but like all other oviraptorosaurs, the manus of *Xingtianosaurus ganqi* preserves three unguals. The large two belong to digit I and II, while the relatively small one belongs to digit III. Although the full digit III is not preserved, the remaining bones are slender, as in other oviraptorosaurs.

**Figure 7:**
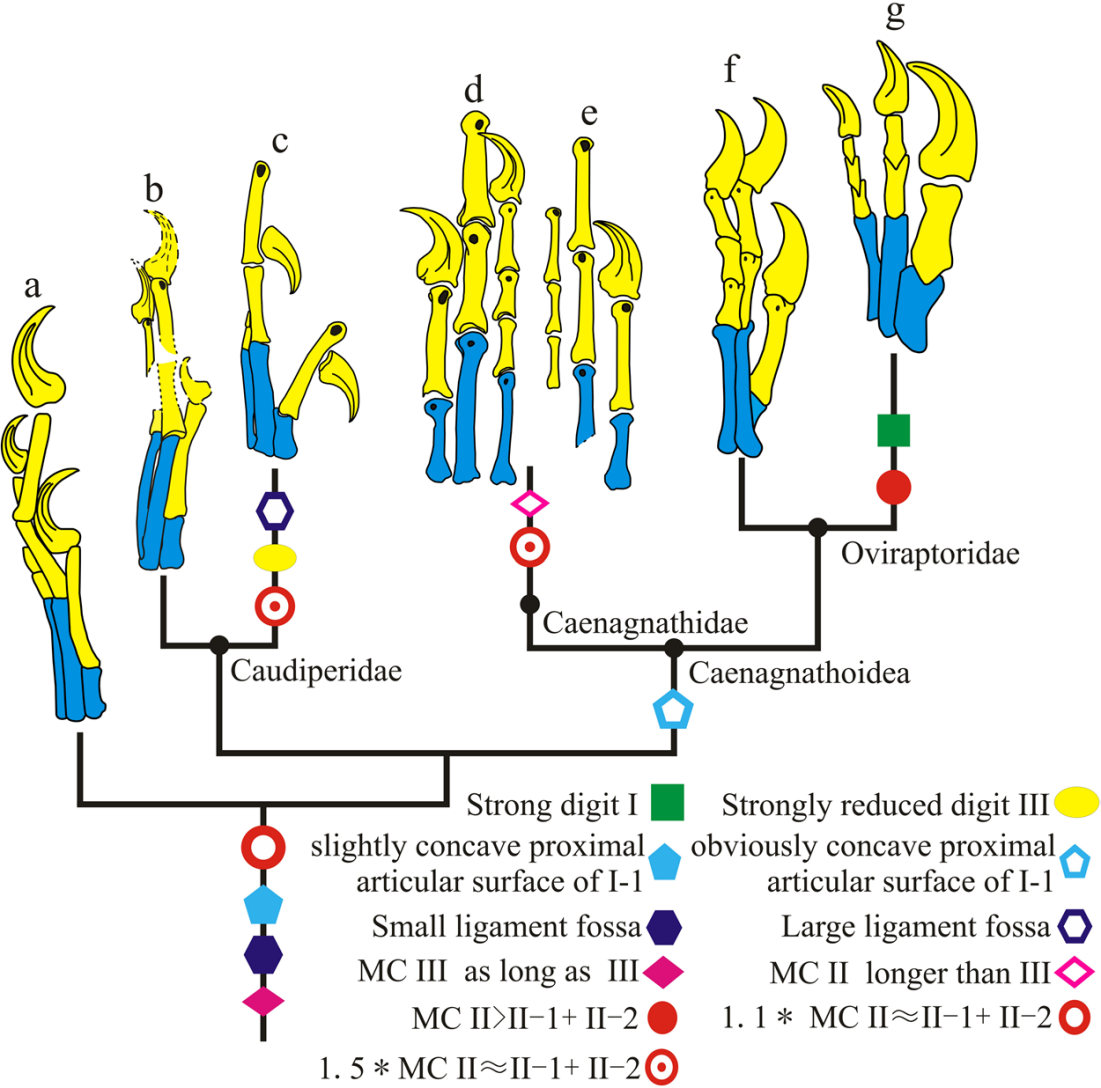
The major changes of oviraptorosaur manus. (**a**) *Protarchaeopteryx*; (**b**) *Xingtianosaurus*; (**c**) *Caudipteryx*; (**d**) *Elmisaurus*; (**e**) *Hagryphus*; (**f**) *Citipati*; (**g**) *Heyuannia*. (**a**) is redrawn from Ji & Ji^47^; (**c**) is redrawn from Zhou *et al*.^6^; (**d**) is redrawn from Zanno & Sampson^25^; (**e**) is redrawn from Osmólska^24^; (**f**) is redrawn from Clark *et al*.^14^; (**g**) is redrawn from Easter^41^. The line drawings are not to scale. (Drawing by R.Q).

The study of the hand of *Xingtianosaurus ganqi* also gives new understanding of the evolution of hand of oviraptorosaurs (Fig. 7). In both Caudipteridae and Caenagnathidae, the combined length of manual phalanx II-1 and II-2 is 1.5 times longer than metacarpal II. But the second digit of *Xingtianosaurus ganqi* is relatively shorter than the combined length of phalanx II-1 and II-2 is about 1.1 times longer than metacarpal II, closer to the ratio of Oviraptoridae, except “Ingeniinae”. This indicates that the primitive condition in Oviraptorosauria is non-ungual phalanges of the second digit subequal in length to metacarpal II. The elongated conditions in Caudipteridae and Caenagnathidae are independently evolved.

Some studies have regarded *Caudipteryx* as a kind of secondarily flightless avian^37–39^ according to some features shared with birds, such as the third manual digit strongly reduced, the propatagium on the forelimb, and the absence of a supracetabular crest of ilium. The features of the manus of *Xingtianosaurus* indicate the specialized hand of *Caudipteryx* is derived from other oviraptorosaurs. In addition, the absence of a supracetabular crest can be found not only in *Caudipteryx* and paraves, but also in many oviraptorosaurs and some non-pennaraptoran coelurosaurs such as *Huaxiagnathus* (Compsognathidae)^40^. A propatagium is existence on forelimb of *Microraptor*^38^, and is not a synapomorphy of avians. These result support *Caudipteryx* as a true dinosaur, rather than a flightless avian.

### Radiale angle evolution in oviraptorosauria

The radiale angle was first proposed as a useful character by Sullivan *et al*.^19^ (Fig. 6a). It is the angle between the articular surface for the radius and semilunate carpal, and it is related to the flexibility of the manus. A large radiale angle is an osteological correlate of a greater range of abduction of the manus. In Coelurosauria, a small radiale angle is the primitive state and it becomes enlarged in maniraptorans. The enlarged radiale angle of maniraptorans means not only a more flexible manus, but also a capability to protect long plumage on their forearm.

Sullivan *et al*.^19^ interpreted the radiale angle in oviraptorosaur as unusually large, even greater than basal Avialae. This is because the oviraptorosaur taxon Sullivan *et al*. chose to represent the entire group is *Caudipteryx*, which has a radiale angle of 76°. To verify the condition of the radiale angle in Oviraptorosauria, we measured other oviraptorosaurs with a well-preserved radiale. Only angles measured from a radiale preserved in the three-dimension or two-dimensional radiale with at least partially exposed articular surface are reliable. The result shows that the radiale angle is 93° in *Hagryphus* (Caenagnathidae), 62° in *Khaan*, 49° in *Machairasaurus* (Oviraptoridae). Thus, all measurements of the radiale angle of oviraptorosaurs are relatively larger than other non-avian maniraptorans. But it should be noted that *Caudipteryx* is an extremely specialized species, and the other measured species are derived, so they do not adequately represent the basal condition of Oviraptorosauria. As a primitive species, the radiale angle of *Xingtianosaurus* is more representative of the basal condition. Its low radiale angle coincides with the evolutionary trend of radiale angle increase in Coelosauria (Fig. 6b). This measurement indicates, like many other characters such as toothless jaws, short nasals, and a rod-like jugal bar^28^, a large radiale angle similar to birds evolved independently in derived oviraptorosaurs.

Most oviraptorosaurs with preserved radiales show a large angle. But the radiale of derived “Ingeniinae” is quite different from other oviraptorosaur groups^21,22,41,42^. Their radiale is small, less than one-third of the size of the semilunate carpal in *Heyuannia* and even less than one-fourth in *Jiangxisaurus*, whereas radiale is larger than half the size of the semilunate carpal in other oviraptorosaurs. The radiale of “Ingeniinae” is quadrilateral, rather than wedge-shaped like in most maniraptorans. The function of the manus in these dinosaurs may be changed from other theropods.

## Methods

The phylogenetic analysis of Coelurosauria is from a large recently published data matrix^34^ (853 characters and 153 taxa). The oviraptorosaurian matrix (252 characters and 39 taxa) is modified from combination of Osmólska *et al*.^1^, Lamanna *et al*.^18^, and Funston & Currie^15^, with 3 added characters as following:

251. Ratio of width of metacarpal III to metacarpal II: more than 0.5 (0); less than 0.5 (1).

252. Ligament pit on manual phalanges: small and dorsally located (0); large and covering most distal end (1).

253. Proximal articular surface of first phalanx of digit I: slightly concave (0); obviously concave (1).

We analyzed both datasets with Tree Analysis Using New Technology (TNT) version 1.1^43^. The coelurosaurian analysis used the “New Technology” search options, with sectorial search, ratchet, tree drift and tree fusion, recovering a minimum tree length in 10 replicates. In oviraptorosaurian analysis, we used traditional search with tree bisection-reconnection (TBR) swapping algorithm with random seeds of 1,000 and 1,000 replicates, saving ten trees per replication.

## Supplementary information

A new caudipterid from the Lower Cretaceous of China with information on the evolution of the manus of Oviraptorosauria
