# Supplementary material for: A new caudipterid from the Lower Cretaceous of China with information on the evolution of the manus of Oviraptorosauria
Source: Sci Rep. 2019 Apr 25;9:6431. doi: 10.1038/s41598-019-42547-6 (PMC6483983; doi:10.1038/s41598-019-42547-6)
Supplement: Supplementary file 1 — A new caudipterid from the Lower Cretaceous of China with information on the evolution of the manus of Oviraptorosauria [file 41598_2019_42547_MOESM1_ESM.docx]

**A new caudipterid from the Lower Cretaceous of China with information on the evolution of the manus of Oviraptorosauria**

**Rui QIU^1, 2, 3^, Xiaolin WANG ^1,2,3^ *, Qiang WANG^1,2^, Ning LI^1, 2^, Jialiang ZHANG^4^, Yiyun MA^5^**

^1^Key Laboratory of Vertebrate Evolution and Human Origin of the Chinese Academy of Sciences, Institute of Vertebrate Paleontology and Paleoanthropology, Beijing, 100044, China

^2^CAS Center for Excellence in Life and Paleoenvironment, Beijing, 100044, China

^3^University of Chinese Academy of Sciences, Beijing, 100049, China

^4^School of Earth Sciences and Resources, China University of Geosciences Beijing, Beijing, 100083, China

^5^College of Life Sciences, Capital Normal University, Beijing, 100048, China

Correspondence and requests for materials should be addressed to X.W. (email: [wangxiaolin@ivpp.ac.cn](mailto:wangxiaolin@ivpp.ac.cn))

Content

1. Supplementary table

2. Supplementary methods.

1. Supplementary table

Measurements (mm) of *Xingtianosaurus ganqi* gen. et sp. nov. (IVPP V13390). Those with * represent estimated values.

| Caudal series | 176.27 |
| --- | --- |
| Scapula | 56.22*/? |
| Sternal plate | 32.74* |
| Humerus | 72.39/? |
| Ulna | 73.46/69,53 |
| Radius | 69.93/63.25 |
| Metacarpal I | 16.57/15.40 |
| Metacarpal II | 40.48/? |
| Metacarpal III | 37.19/? |
| Manus phalanx I-1 | 25.45/? |
| Manus phalanx II-1 + II-2 | 44.75/? |
| Manus phalanx II-3 | 21.19/19.74* |
| Manus phalanx III-4 | 15.40/15.63 |
| Ilium | 89.84* |
| Pubis | 106.96* |
| Ischium | 54.56* |
| Femur | 126.95/120.36 |
| Tibia | 171.25/? |
| Metatarsal I | 9.79/10.17 |
| Metatarsal II | 80.41/78.49 |
| Metatarsal III | 89.65/90.24 |
| Metatarsal IV | 82.76/82.87 |
| Metatarsal V | 25.60/? |
| Pedal phalanx I-1 | ?/19.03 |
| Pedal phalanx I-2 | ?/14.58 |
| Pedal phalanx II-1 | 28.34/27.84 |
| Pedal phalanx II-2 | 22.55/23.80 |
| Pedal phalanx III-1 | 26.99/27.01 |
| Pedal phalanx III-2 | 20.88/20.68 |
| Pedal phalanx III-3 | 20.32/21.38 |
| Pedal phalanx III-4 | 17.16/15.04 |
| Pedal phalanx IV-1 | 17.31/17.62 |
| Pedal phalanx IV-2 | 14.47/14.30 |
| Pedal phalanx IV-3 | 12.66/12.83 |
| Pedal phalanx IV-4 | 15.97/14.72 |
| Pedal phalanx IV-5 | 15.96/15.84 |

2. Supplementary methods.

2.1 Character list of Oviraptorosauria

1. Ratio of the preorbital skull length to the basal skull length: 0.6 or more (0); less than 0.6 (1)
2. Pneumatized crest-like prominence on the skull roof: absent (0) present (1)
3. Ratio of the width (across premaxilla-maxilla suture) of the snout to its length: less than 0.3 (0); 0.3-0.4 (1); more than 0.4 (2)
4. Ratio of the length of the tomial margin of the premaxilla to the premaxilla height (ventral to the external naris): 1.0-1.4 (0); more than 1.7 (1); 0.7 or less (2)
5. Inclination of the anteroventral margin of the premaxilla relative to the horizontally positioned ventral margin of the jugal: vertical (0); posterodorsal (1); anterodorsal (2)
6. Ventral projection of the premaxilla below the ventral margin of the maxilla: absent (0); small (1); significant (2)
7. Share of the premaxilla (ventral) in the basal skull length: 0.10 or less (0); 0.12 or more (1)
8. Pneumatization of the premaxilla: absent (0); present (1)
9. Ratio of the length of the maxilla (in lateral view) to the basal skull length: 0.4-0.7 (0); less than 0.4 (1)
10. Subantorbital portion of the maxilla: not inset medially (0); inset medially (1)
11. Palatal shelf of the maxilla with two longitudinal ridges and a tooth-like ventral process: absent (0); present (1)
12. Ventral margins of maxilla and jugal margins: form a straight line (0); the ventral margin of the maxilla slopes anteroventrally, its longitudinal axis at an angle of ca. 120° to the longitudinal axis of the jugal (1)
13. Rim around antorbital fossa: well pronounced (0); poorly delimited (1)
14. Antorbital fossa: bordered anteriorly by the maxilla (0); bordered anteriorly by the premaxilla (1)
15. Accessory maxillary fenestrae: absent (0); at least one accessory fenestra present (1)
16. Nasal along midline: longer than frontal (0); shorter than or as long as the frontal
17. Nasals: separate (0); fused (1)
18. Subnarial process of the nasal: long (0); short (1)
19. Shape of the narial opening: longitudinally oval (0); teardrop-shaped, slightly longer than wide much longer than wide (1)
20. Nasal recesses: absent (0); present (1)
21. External naris position relative to the antorbital fossa naris and fossa: widely separated (0); posterior margin of the naris reaching the fossa (1) overlapping anterodorsally most of the fossa (2)
22. Ventral margin of the external naris: at the level of the maxilla (0); dorsal to the maxilla (1)
23. Prefrontal: present (0); absent or fused with the lacrimal (1)
24. Lacrimal shaft: not projecting outward beyond the orbital plane and lateral surface of the snout (0); the middle part of the shaft projecting laterally to form a flattened transverse bar in front of the eye (1)
25. Lacrimal recesses: absent (0); present (1)
26. Ratio of the length of the orbit to the length of the antorbital fossa: 0.7-0.9 (0); 1.2 or more (1)
27. Ratio of the length of the parietal to the length of the frontal: 0.6 or less (0); 1.0 or more (1)
28. Pneumatization of the skull roof bones: absent (0); present (1)
29. Sagittal crest along the interparietal contact: absent (0); present (1)
30. Supratemporal fossa: invading the frontal (0); not invading the frontal (1)
31. Infratemporal fenestra: dorsoventrally elongate, narrow anteroposteriorly (0); subquadrate, its anteroposterior length comparable to the orbital length (1)
32. Pneumatization of the squamosal: absent (0); present (1)
33. Cotyle-like incision on the ventrolateral margin of the squamosal (for reception of the dorsal end of the ascending process of the quadratojugal): absent (0); present (1)
34. Ventral ramus of the jugal deep dorsoventrally and flattened lateromedially (0); shallow dorsoventrally or rod-shaped (1)
35. Jugal process of the postorbital: not extending ventrally below two-thirds of the orbit height (0); long, extending ventrally close to the base of the postorbital process of the jugal (1)
36. Postorbital process of the jugal: posterodorsally inclined (0); perpendicular to the ventral ramus of the jugal (1); absent (2)
37. Jugal-postorbital contact: present (0); absent (1)
38. Quadratojugal process of the jugal in lateral view: forked (0); not forked (1); fused with the quadratojugal (2)
39. Quadratojugal-squamosal contact: absent (0); present (1)
40. Ascending (squamosal) process of the quadratojugal: bordering ca. the ventral half, or less, of the infratemporal fenestra (0); bordering the ventral two-thirds or more of the infratemporal fenestra (1); absent (2)
41. Angle between the ascending and jugal processes of the quadratojugal: ca. 90° (0); less than 90° (1)
42. Quadrate process of the quadratojugal: well developed, extending posteriorly or posteroventrally beyond the posterior margin of the ascending process (0); not extending beyond the posterior margin of the ascending process (1)
43. Dorsal part of the quadrate: erect (0); bent backward (1)
44. Otic process of the quadrate: articulating only with the squamosal (0); articulating with the squamosal and the lateral wall of the braincase (1)
45. Pneumatization of the quadrate: absent (0); present (1)
46. Lateral accessory process on the distal end of the quadrate for articulation with the quadratojugal: absent (0); present (1)
47. Lateral cotyle for the quadratojugal on the quadrate: absent (0); present (1)
48. Mandibular condyles of quadrate: posterior to the occipital condyle (0); in the same vertical plane as the occipital condyle (1); anterior to the occipital condyle (2)
49. Nuchal transverse crest: pronounced (0); not pronounced (1)
50. Occiput position in relation to the ventral margin of the jugal-quadratojugal bar: about perpendicular (0); inclined anterodorsally (1)
51. Paroccipital process: directed laterally (0); directed ventrally (1)
52. Foramen magnum: smaller than or equal in size to the occipital condyle (0); larger than the occipital condyle (1)
53. Basal tubera: modestly pronounced (0); well developed, widely separated (1)
54. Pneumatization of the basisphenoid: weak or absent (0); extensive (1)
55. Basipterygoid processes: well developed (0); strongly reduced (0); absent (1)
56. Parasphenoid rostrum: horizontal or anterodorsally directed (0); sloping anteroventrally (1)
57. Depression in the periotic region: absent (0); present (1)
58. Pneumatization of the periotic region: absent or weak (0); extensive (1)
59. Quadrate ramus of the pterygoid: distant from the braincase wall (0); overlapping the braincase (1)
60. Pterygoid basal process for contact with the basisphenoid: absent (0); present (1)
61. Ectopterygoid position: lateral to the pterygoid (0); anterior to the pterygoid (1)
62. Ectopterygoid contacts with the maxilla and lacrimal: absent (0); present (1)
63. Ectopterygoid: short anteroposteriorly with a hook-like jugal process (0); elongate, shaped like a Viking ship, without a hook-like process (1)
64. Massive pterygoid-ectopterygoid longitudinal bar: absent (0); present (1)
65. Palate extending below the cheek margin: absent (0); present (1)
66. Palatine: tetraradiate or trapezoidal (0); triradiate, without a jugal process (1); developed in horizontal, longitudinal, and transverse planes perpendicular to each other (2)
67. Pterygoid wing of the palatine: dorsal to the pterygoid (0); ventral to the pterygoid
68. Maxillary process of the palatine: shorter than the vomeral process (0); longer than the vomeral process (1)
69. Vomer: distant from the parasphenoid rostrum (0); approaching or in contact with the parasphenoid rostrum (1)
70. Suborbital (ectopterygoid-palatine) fenestra: well developed (0); closed or reduced
71. Jaw joint: distant from the midline of the skull (0); close to the skull midline (1)
72. Movable intramandibular joint: present (0); suppressed (1)
73. Mandibular symphysis: loose (0); tightly sutured (1); fused (2)
74. Extended symphyseal shelf at the mandibular symphysis: absent (0); present (1)
75. Downturned symphyseal portion of the dentary: absent (0); present (1)
76. U-shaped mandibular symphysis: absent (0); present (1)
77. Ratio of the length of the retroarticular process to the total mandibular length: less than 0.05 or the process absent (0); ca. 0.10 (1)
78. Dentary: elongate (0); proportionally short and deep, with maximum depth of dentary between 25% and 50% of dentary length (with length measured from the tip of the jaw to the end of the posterodorsal process) (1); extremely short and deep, with maximum depth 50% or more of dentary length (2)
79. Ratio of the height of the external mandibular fenestra to the length of the fenestra: 0.2-0.5 (0); 0.7-1.0 (1); fenestra absent (2)
80. Ratio of the length of the external mandibular fenestra to total mandibular length: 0.15-0.20 (0); not more than 0.10 or fenestra absent (1); 0.25 or more (2)
81. Process of the surangular dividing the external mandibular fenestra: absent (0); short and broad elongate and spike-like (1)
82. Co-ossification of the articular with the surangular: absent (0); present (1)
83. Mandibular rami in dorsal view: straight (0); laterally bowed at midlength (1)
84. Anterodorsal margin of dentary in lateral view: straight (0); concave (1); broadly concave (2)
85. Posterior margin of the dentary: incised, producing two posterior processes (0); oblique (1)
86. Posterodorsal process of the dentary: long and shallow present (0); absent (1)
87. Posteroventral process of the dentary: shallow and long, extending posteriorly at least to the posterior border of the external mandibular fenestra absent (0); present (1)
88. Coronoid process: posteriorly positioned and vertically projected (0); anteriorly positioned, near the midpoint of the jaw, with a medially hooked apex (1)
89. Surangular foramen: present (0); absent (1)
90. Mandibular articular facet for the quadrate: comprising the surangular and the articular (0); formed exclusively of the articular (1)
91. Mandibular articular facet for the quadrate: with one or two cotyles (0); convex in lateral view, transversely wide (1)
92. Position of the quadrate articular surface relative to the level of the adjoining dorsal margin of the mandibular ramus: ventral (0); moderately elevated, quadrate articulation grades smoothly into remainder of mandible (1); highly elevated, anterior and posterior margins of quadrate articulation at nearly right angles to remainder of mandible (2)
93. Anterior part of the prearticular: deep, approaching the dorsal margin of the mandible (0); shallow, strap-like, not approaching the dorsal mandibular margin (1)
94. Splenial: subtriangular, approaching the dorsal mandibular margin (0); strap-like, shallow, not approaching the margin (1)
95. Mandibular adductor fossa: anteriorly delimited, occupying the posterior part of the mandible (0); large, anteriorly and dorsally extended, not delimited anteriorly (1)
96. Coronoid bone: well developed (0); strongly reduced (1); absent (2)
97. Premaxillary teeth: present (0); absent (1)
98. Maxillary tooth row: extends at least to the level of the preorbital bar (0); does not reach the level of the preorbital bar (1); maxillary teeth absent (2)
99. Dentary teeth: present (0); absent from tip of jaw but present posteriorly (1); absent (2)
100. Number of cervicals (excluding cervicodorsal): not more than 10 (0); more than 10 (1)
101. Anterior articular facets of the centra in the anterior postaxial cervicals: not inclined or only slightly inclined (0); strongly inclined posteroventrally, almost continuous with the ventral surfaces of the centra (1)
102. Centra of the anterior cervicals: not extending posteriorly beyond their respective neural arches (0); extending posteriorly beyond their respective neural arches (1)
103. Epipophyses on the postaxial cervicals: in the form of a low crest or rugosity (0); prong-shaped (1)
104. Cervical ribs in adults: loosely attached to the vertebrae (0); firmly attached (1); fused (2)
105. Shafts of cervical ribs: longer than their respective centra (0); not longer than their respective centra (1)
106. Lateral pneumatic fossae ("pleurocoels") on the dorsal centra: absent (0); present (1)
107. Ossified uncinate processes on the dorsal ribs: absent (0); present (1)
108. Number of vertebrae included in the synsacrum in adults: not more than 5 (0); 6 (1); 7-8 (2)
109. Sacral spines in adults: unfused (0); fused (1)
110. Lateral pneumatic fossae on the sacral centra: absent (0); present (1)
111. Transition point on the caudals: absent (0); present (1)
112. Number of caudals with transverse processes: 15 or more (0); fewer than 15 (1)
113. Lateral pneumatic fossae on the caudal centra: absent (0); present at least in the anterior part of the tail (1)
114. Neural spines: confined to at least 23 anterior caudals (0); at most 16 anterior caudals (1)
115. Number of caudals: more than 35 (0); 30 or fewer (1)
116. Posterior caudal prezygapophyses: overlapping less than half of the centrum of the preceding vertebra (0); overlapping at least half of the centrum of the preceding vertebra (1)
117. Hypapophyses in the cervicodorsal vertebral region: absent (0); small (1); prominent (2)
118. Posterior hemal arches: deeper than long (0); longer than deep (1)
119. Ratio of the length of the scapula to the length of the humerus: 0.8-1.1 (0); 1.2 or more (1); 0.7 or less (2)
120. Acromion: projecting dorsally (0); projecting anteriorly (1); everted laterally (2)
121. Posteroventral process of the coracoid: absent or short, not extending beyond the glenoid diameter (0); long, posteroventrally extending beyond the glenoid (1)
122. Orientation of the glenoid on the pectoral girdle: posteroventral (0); lateral (1)
123. Deltopectoral crest: low, its width equal to, or smaller than, the shaft diameter (0); expanded, wider than the shaft diameter (1)
124. Extent of the deltopectoral crest (measured from the humeral head to the apex): about the proximal third of the humerus length or less (0); ca. 40%-50% of the humerus length (1)
125. Shaft of the ulna: straight (0); bowed, convex posteriorly (1)
126. Ratio of the length of the radius to the length of the humerus: 0.80 or less (0); 0.85 or more (1)
127. Combined length of manual phalanges III-1 and III-2: greater than the length of phalanx III-3 (0); less than or equal to the length of phalanx III-3 (1)
128. Ratio of the length of metacarpal I to the length of metacarpal II: 0.5 or more (0); less than 0.5 (1)
129. Proximal margin of metacarpal I in dorsal view: straight, horizontal (0); angled due to a medial extent of carpal trochlea (1)
130. Metacarpal II relative to metacarpal III: shorter (0); longer (1); subequal (2)
131. Ratio of the length of metacarpal II to the length of the humerus: 0.4 or less (0); more than 0.4 (1)
132. Ratio of the length of the manus to the length of the humerus plus the radius: 0.50-0.65 (0); more than 0.65 (1); less than 0.50 (2)
133. Ratio of the length of the manus to the length of the femur: 0.3-0.6 (0); more than 0.7 (1)
134. Ratio of the length of the humerus to the length of the femur: 0.50-0.69 (0); 0.70 or more (1)
135. Dorsal margins of opposite iliac blades: well separated from each other (0); close to or contacting each other along their medial sections (1)
136. Dorsal margin of the ilium along the central portion of the blade: straight (0); arched (1)
137. Preacetabular process of the ilium relative to the postacetabular process (lengths measured from the center of the acetabulum): shorter or equal (0); longer (1)
138. Preacetabular process: not expanded or weakly expanded ventrally below the level of the dorsal acetabular margin (0); expanded ventrally well below the level of the dorsal acetabular margin (1)
139. Morphology of the ventral margin of the preacetabular process: cuppedicus fossa absent, margin transversely narrow (0); cuppedicus fossa or a wide shelf present (1); margin flat, wide at least close to the pubic peduncle (2)
140. Anteroventral extension of the preacetabular process: absent (0); with rounded tip (1); hook-like (2)
141. Posterior end of the postacetabular process: truncated or broadly rounded (0); narrowed or acuminate (1)
142. Anteroposterior length of the pubic peduncle: about the same as that of the ischial peduncle (0); distinctly greater than that of the ischial peduncle (1)
143. Dorsoventral extension of the pubic peduncle: level with the ischial peduncle (0); deeper than the ischial peduncle (1)
144. Ratio of the length of the ilium to the length of the femur: 0.50-0.79 (0); 0.80 or more (1)
145. Pelvis: propubic (0); mesopubic (1); opisthopubic (2)
146. Pubic shaft: straight (0); concave anteriorly (1)
147. Pubic foot: anterior and posterior processes about equally long (0); anterior process absent or shorter than the posterior process (1); anterior process longer than the posterior process (2)
148. Posterior margin of the ischial shaft: straight or almost straight (0); distinctly concave (1)
149. Greater trochanter of the femur: weakly separated, or not separated, from the femoral head (0); distinctly separated from the femoral head (1)
150. Anterior and greater trochanters: separated (0); contacting (1)
151. Dorsal extremity of the anterior trochanter: well below the greater trochanter (0); about level with the greater trochanter (1)
152. Fourth trochanter: well developed (0); weakly developed or absent (1)
153. Adductor fossa and the associated anteromedial crest on the distal femur: weak or absent (0); well developed (1)
154. Distal projection of the fibular condyle of the femur beyond the tibial condyle: absent (0); present (1)
155. Ascending process of the astragalus: as tall as wide across the base (0); taller than wide (1)
156. Distal tarsals: not fused with the metatarsus (0); fused with the metatarsus (1)
157. Proximal coossification of metatarsals II-IV: absent (0); present (1)
158. Arctometatarsus: absent (0); present, but only proximal-most extreme of metatarsal III obscured from anterior view in articulated metatarsus (1); present, proximal ~half of metatarsal III obscured from anterior view in articulated metatarsus (2)
159. Length of metatarsal I: constituting more than 50% of metatarsal II length (0); less than 50% of metatarsal II length (1); metatarsal I absent (2)
160. Ratio of the maximum length of the metatarsus to the length of the femur: 0.4-0.6 (0); ca. 0.3 (1); 0.7-0.8 (2)
161. Crenulated tomial margin of the premaxilla: absent (0); present (1)
162. Frontals: flat or weakly arched, not strongly projecting above orbit in lateral view (0); strongly arched, projecting well above orbit in lateral view to contribute to nasal-frontal crest (1)
163. Exoccipital: short, weakly projecting (0); strongly projects ventrally beyond squamosal in lateral view, approaching ventral end of the quadrate (1)
164. Dentary posterodorsal ramus: straight or weakly curved (0); strongly bowed dorsally (1)
165. Prominent symphyseal process on posteroventral surface of dentary symphysis: absent (0); present (1)
166. Dentary: anteroventral margin in lateral view straight or weakly downturned (0); strongly downturned (1)
167. Lateral surface of dentary: smooth (0); bearing a deep fossa, sometimes with associated pneumatopore (1)
168. Angular: contributes extensively to the border of the external mandibular fenestra (0); largely excluded from the border of the external mandibular fenestra by the surangular (1)
169. Surangular with an anteroposteriorly elongate flange on the ventral edge: absent (0); present (1)
170. External mandibular fenestra: elongate (0); height subequal to length (1)
171. Dentary contribution to external mandibular fenestra: no more than 50% length of dentary (0); exceeds 50% length of dentary (1)
172. Metacarpal I expanded ventrally to cover ventral surface of metacarpal II: absent (0); present (1)
173. Unguals of manual digits II and III: strongly curved (0); weakly curved (1)
174. Manual phalanx I-1: slender (0); more robust than II-1 (1); more than 200% diameter of II-1 (2)
175. Manual phalanx III-3: longer than phalanx III-2 (0); does not exceed length of III-2 (1)
176. Manual phalanx II-2: longer than II-1 (0); subequal to or slightly shorter than II-1 (1); distinctly shorter than II-1 (2)
177. Manual digit II: elongate, with combined lengths of manual phalanges II-1 and II-2 longer than metacarpal II (0); combined lengths of manual phalanges II-1 and II-2 subequal to metacarpal II (1)
178. Ischium strongly bent posteriorly at midshaft distal end forms an angle of at least 60 with proximal end absent (0); present (1)
179. Metatarsus: elongate (0); short, length does not exceed 300% of proximal width
180. Ilium: tall (0); low and anteroposteriorly elongate, height less than 25% of length (1)
181. Anterior blade of ilium shallower than posterior blade: absent (0); present (1)
182. External naris: placed anteriorly (0); extends posteriorly, with posterior end lying above antorbital fenestra (1)
183. Premaxillae, nasal processes anteroposteriorly expanded and mediolaterally compressed to form a bladelike internarial bar: absent (0); present (1)
184. Dentary, anterodorsal tip of beak: projects upwards (0); anterodorsally, tip of beak projecting at an angle of 45° or less relative to the ventral margin of the symphysis (1)
185. Dentary symphysis with interior surface bearing vascular grooves and associated foramina: absent (0); present (1)
186. Dentary symphysis bearing an hourglass-shaped ventral depression: absent (0); present (1)
187. Meckelian groove terminates on the inside of the dentary (0); on the ventral surface of the symphysis
188. Lingual triturating shelf of dentary: absent (0); present (1)
189. Dentary, symphyseal ridges inside the tip of the beak: absent (0); present but weakly developed (1); present and well developed (2)
190. Dentary, lingual ridges inside the lateral occlusal surface of beak: absent (0); present (1)
191. Posteroventral process of dentary: straight (0); bowed ventrally (1)
192. Dentaries pneumatized: absent (0); present (1)
193. Dentary: participates in dorsal border of the external mandibular fenestra (0); excluded from dorsal border of external mandibular fenestra by anterior extension of the surangular (1)
194. Dentary: participates in ventral border of external mandibular fenestra (0); excluded from ventral border of external mandibular fenestra by anterior extension of the angular (1)
195. Surangular and angular divided by posterior extension of the external mandibular fenestra: absent (0); present (1)
196. Posterior end of the surangular: deep (0); shallow, subequal to or shallower than angular (1)
197. Surangular: deep anteriorly (0); strap-like (1)
198. Retroarticular process extends: posteriorly (0); posteroventrally (1); posterolaterally (2)
199. Metacarpal I: proportionately broad (0); long and slender, diameter 20% of length (1)
200. Manual phalanx I-1: longer than II-2 (0); subequal to II-2 (1); shorter than II-2 (2)
201. Ischiadic peduncle of pubis with prominent medial fossa: absent (0); present (1)
202. Ischium, obturator process located: distally (0); at midshaft of ischium (1)
203. Anterior margin of obturator process: straight or convex (0); distinctly concave (1)
204. Accessory trochanter of femur: weakly developed (0); prominent, subrectangular flange or finger-like process (1)
205. Metatarsal III: with an ovoid or subtriangular cross section (0); anteroposteriorly flattened, with a concave posterior surface (1)
206. Paroccipital process: elongate and slender, with dorsal and ventral edges nearly parallel (0); short and deep with convex distal end (1)
207. Mandibular articulation surface: as long as ventral end of quadrate (0); twice or more as long as quadrate surface, allowing anteroposterior movement of mandible (1)
208. Scars for interspinous ligaments in dorsal vertebrae terminate: at apex of neural spine (0); ventral to apex of neural spine (1)
209. Sternum, distinct lateral xiphoid process posterior to costal margin: absent (0); present (1)
210. Anterior edge of sternum: grooved for reception of coracoids (0); without grooves (1)
211. Deltopectoral crest: large and distinct, proximal end of humerus quadrangular in anterior view (0); less pronounced, forming an arc rather than being quadrangular (1)
212. Ischium: more than two-thirds of pubis length (0); two-thirds or less of pubis length (1)
213. Lateral ridge of femur: absent or represented (0); only by faint rugosity distinctly raised from shaft, mound-like (1)
214. Surangular, distinct groove on dorsal surface: present (0); absent (1)
215. Vomer, position: level with other palatal elements (0); ventral to other palatal elements (1)
216. Calcaneum: excludes astragalus from reaching lateral margin of tarsus (0); small process of astragalus protrudes through a circular opening in edge of calcaneum to reach lateral margin of tarsus (1)
217. Depression on lateral surface of dentary immediately anterior to external mandibular fenestra: absent (0); present (1)
218. Groove on ventrolateral edge of angular to receive posteroventral branch of dentary: absent (0); present (1)
219. Posteroventral branch of dentary twisted so that lateral surface of branch faces somewhat ventrally: absent (0); present (1)
220. Premaxilla, large, presumably pneumatic foramen at anteroventral corner of narial fossa: absent (0); present (1)
221. Accessory opening at anterodorsal extreme of snout: absent (0); present (1)
222. Development of symphyseal shelf of mandible: limited, anteroposterior length of mandibular symphysis (as measured on midline) less than 20% total anteroposterior length of mandible (0); intermediate, length of symphysis greater than 20% but less than 25% length of mandible (1); extensive, length of symphysis greater than 25% mandibular length (2)
223. Prominent flange or shelf arising from lateral surface of dentary: absent (0); present (1)
224. Base of retroarticular process: considerably wider mediolaterally than tall (0); dorsoventrally approximately as wide as tall (1); considerably taller than wide (2)
225. Posterior-most caudal vertebrae fused, forming a pygostyle-like structure: absent (0); present (1)
226. Humeral shaft: straight or nearly straight (0); strongly bowed laterally (1)
227. Proximodorsal "lip" on manual unguals: weak (i.e., continuous or nearly continuous with remainder of dorsal surface of ungual) and/or absent (0); prominent ("set off" from remainder of dorsal surface by distinct change in slope immediately distal to "lip") (1)
228. Pubic process of ischium, "hooked" anterodistal extension: absent (0); present (1)
229. Posterodistal margin of obturator process: straight (0); distinctly concave, apex of obturator process angled distally (1)
230. Proximolateral edge of metatarsal IV attenuated into pointed process: absent (0); present (1)
231. Frontal anteriorly divided by slot for nasal and possibly lacrimal: absent (0); present (1)
232. Infradiapophyseal infraprezygapophyseal and infrapostzygapophyseal fossae on cervical and dorsocervical vertebrae: one or more absent (0); all three present (1)
233. Ratio of minimum shaft diameter of manual phalanx II-1 to minimum shaft diameter of metacarpal: II >1 (0); <1 (1)
234. Ratio of minimum shaft diameter to length of manual phalanx II-2: >0.10 (0); <0.10 (1)
235. Ratio of the length of the metatarsus to the length of the tibia: <0.5 (0); >0.5 (1)
236. Tibia ratio of the transverse width of the distal condyles to the length: 0.20 or greater (0); <0.20 (1)
237. Ratio of minimum transverse width to length of tarsometatarsus: >0.20 (0); <0.20 (1)
238. Fusion of distal tarsals III and IV at maturity: absent (0); present (1)
239. 'Hook-like' posterodorsal process of distal tarsal IV: absent (0); present (1)
240. Posterior protuberance on proximal end of tarsometatarsus caused by coossification of distal tarsals III and IV, plus MT II, III and IV: absent (0); present (1)
241. Anterior margin of metatarsal V in lateral view: straight or slightly curved (0); tightly curved (1)
242. Concavity on posterior surface of tarsometatarsus in cross section: absent or shallow (0); prominent and deep (1)
243. Sharp cruciate ridges on posterior surface of metatarsal III: only one sharp longitudinal ridge or no ridges (0); sharp medial longitudinal ridge continuous with lateral postcondylar ridge and sharp lateral longitudinal ridge continuous with medial postcondylar ridge forming a chiasmata. Ridges are separated from each other by longitudinal sulcus (1)
244. Posteromedial and posterolateral ridges of mt II and IV respectively: weakly developed do not extend past posterior extent of distal condyle (0); well developed extend posteriorly past distal condyle (1)
245. Distal ends of shafts of metatarsals II and IV: both straight (0); metatarsal II medially deflected metatarsal IV straight (1); metatarsal II straight metatarsal IV laterally deflected (2)
246. Ratio of transverse width to anteroposterior length of distal condyle of metatarsal III: <1 (0); >1 (1)
247. External mandibular fenestra: expanded anteriorly anteriorly (0); constricted by posteroventral ramus of dentary (1)
248. Articular ridge of mandible: low, less than 25% (0); as tall as long high, more than 25% as tall as long (1)
249. Transverse groove between flexor tubercle and proximal articular surface of manual ungual I-2: absent (0); present (1)
250. Cnemial crest of tibia greatly enlarged such that the proximal articular surface of the tibia is longer anteroposteriorly than wide mediolaterally: absent (0); present (1)
251. Ratio of width of metacarpal III to metacarpal II: more than 0.5 (0); less than 0.5 (1).
252. Ligament pit on manual phalanges: small and dorsally located (0); large and covering most distal end (1).
253. Proximal articular surface of first phalanx of digit I: slightly concave (0); obviously concave (1)

2.2 Datamatrix of Oviraptorosauria

Herrerasaurus_ischigualastensis

00000000000000000000000000000000000000000000000000000000000000000000000000000000000000000000000000000010?0000000000100?0000101??0000000000000000100000000000000000000?000?0000111010100??000??0000101??00??0000???00?1000?000000?00000???????000?0000?000?000

Velociraptor_mongoliensis

000100000000101001010010100010000000001000000001000010000000000000000000000000010000110000000000010010110111101101011101?110101111011100001111112010011100010011000000000?0000003011000?0000??00100001000000000110011?000?00000000000000?????00000000?000?101

Archaeopteryx_lithographica

000?10000000101101001100010001?10002110?0?1??0020????00????0000001?1???100001021000011?0100000020100??0101?00?110110012111001111121011001010111020101?0100?1101200?0000?0?0?00003001000?0000??001?000000?010010???111??00?00000?0000?00??????00000000?000?101

Incisivosaurus_gauthieri

100110101000011?0111210011?01?00?0000?10010?0??200111?1?10?01??111?0101?210111020??00011??11?111011?????????????????????????????????????????????????????????????00000000100??????????00100000000000000???????1???????00???01000???????0???????????????000????

Caudipteryx_zoui

10?110?01??0?1110?0?21?01?10???0110001110100??????????????????0????????0??1111?2???0001??????11?0220?????010????0?1??10???1011?1020100?001220110?021100???1000121??0???0??0?00?031000?000?00000000?01000?0010???0101????0?00000?0000000?0??????000000?000?110

Avimimus_portentosus

??????????0???????????????10110??1?2121201??00?101111?1???????????????0?1??111??01?????11000???21?21000110?110??0???2?02100111?1?????00000?210?10020101001111222100010000?0???????00??00?00????0??0000??0??10110??11110????????0?0?000010?????1000000?00?????

Microvenator_celer

????????????????????????????????????????????????????????????????????????1111?2????11001???????????2???0??1?????????0????1?1011??1????0???112?11?112??011101????????0101???0011?????00??0000000?0??????0?1??1???0??1?0???1?????0??00??????????????????????????

Oviraptor_philoceratops

1??????????1?11?????21????????1??1000???0???????????????????????1?????????1?12122??10011???1??1??22???0??1????????????1???101101?211?????????????????????????????0???001001000000??0??1????????????????0??????????0??11?0?0???0??00???0???????????????000?00?

Rinchenia_mongoliensis

11122?0111110111???1211111111?111101011100111111?1111121?11111111211111111111212?0110011111111121221110011?1010010102?12101000???????01101121101????111111??????1101?001011????????00111??????00000001????????1?10???1110?0?0?0???????????????????????000????

Citipati_osmolskae

11?2221?1111011?11112111111?111?1111011111??????111????11?1?11111????1?11?1112122011001111111111122???????1????????????????0?101?2??????????????????????????????11011001011?01010?0??1100000000000000100?????010100?01110?001000100?0?0??????00?0??00?000?001

Zamyn_Khondt_oviraptorid

1122221111110111111121111111111111110111111111111111112111111111121111111111121220110011111111121221110111?101001010210210101101121110100121100111211111111000101101?001011?0000010??110?0000000000?0100???00?1?100??11???001?0??00??00???????????????000?00?

Khaan_mckennai

1022111?1111011?112121111111111?1100011100?111?111111??0??1?????1??????11111121210110011111111??1221110??1????00?0102??210??1101021110?000110001?1211111?1100010100110010010010100111110?000?001000001000000?01?1100?1?10101000?0000100??????00000000?000?001

Conchoraptor_gracilis

10121111111101111121211111111111110001110?1111111?111121111111111211111111111?12?0110011111111121221110111?20100101011121011110012111010002100011121111111100010101?????????110???1??11????????????????0??????11?00?011?0?0000011?0??000?????00000000?000?00?

Machairasaurus_leptonychus

??????????????????????????????????????????????????????????????????????????????????????????????????2???????????????????????????0012?????????????????????????????????????????111110?????????????????????00??????????????????????????1???????????????????????101

Nemegtomaia_barsboldi

1112011111110111111121011111111?110001011011?111111?1??11?1111111211111111111212?11100111111?1121221110211?201????????????????????????10002??00?1????11?????????10111101011?12?11??1111000000010000001000??0??1???1??11?1?1?0001?000??00??????????????000?011

Heyuannia_huangi

?????????????????????????????????1????????????????????????????????????????1??2121??1001????1??????21???21?12????1?????121?11000002?20???01?1100111?1???????????????1?1?1011112?2101?1?????????0?00000100?0?0?0101?00?1?11?0???0?00001????????000000000000?001

Ingenia_yanshini

???????1???????????????11?????1?11000111?0111111???111??11?1??111?1???1111111212?011001111111112122?110011?20100101010121011000012020010012100011121111111100010?011?10101111212101111100000000000000100?0000?1?100001?1??0???0?000010?00000000000000?000?011

Gigantoraptor_erlianensis

??????????????????????????????????????????????????????????????????????1121111202011100111112????1?2???????????0010?0??00?00000?11?1??1???????????1??1111111000?0???0001010000??????????0???0001001110100???0??????1?????110??112?10??????????010?000??011????

Caenagnathasia_martinsoni

????????????????????????????????????????????????????????????????????????2101?1????120?????????????2?1002?1?0?1??????????????????????????????????????10?1???????????0001???0????????????011?111?1??????????????????????????????0????????0?????????????????????

Caenagnathus_collinsi

??????????????????????????????????????????????????????????????????????10210111020112001111111112??2?????????????1???????????????????????????????????111101?001?????00010101?1??????????110112101111111?????1????????01?0010??201??1????????????0???0??000????

Leptorhynchos_elegans

???????????????????????????????????????????????????????????????????????12111?1????1200???????1????2????????????????????????????????????????????????????????1111????0001???0???????0????0111121?10???????????1???????????0?????0??????1?????111100111111??1???

Elmisaurus_rarus

?????????????????????????????0????????????????????????????????????????????????????????????????????????0??10?????????2????1?????011??????????????????11?1???1111??0?????????000?12?0???????????????????11????1?????????????????????1??11111111111011120??11101

Apatoraptor_pennatus

?????????????????????????????????????????????????????????????????0?1???1211111020111001111111112??211002111?????????2?0111001001110111???101???????????101?????????00010100010?10??????01?11?11111111210??????1?101?11??011??101?01????111????????????100110?

Chirostenotes_pergracilis

??????????????????????????????????????????????????????????????????????11211111020112001111121112??2????????111??????????10?????0????1??11??21101???111?1?1100110???00010100?100121000??01111211111111112?1111????????1??010???01??1000??111100001100100110101

Hagryphus_giganteus

??????????????????????????????????????????????????????????????????????????????????????????????????????????????????????????????0011?????????????????????????????????????????000012??????????????????????2??????????????????????????1?????00??????????????1?101

Anzu_wyliei

?1??101100?1100??????????????????0?000??????1011??100?1??1??0?011?????1121011?020112001111111?121221110211?1?10010?02?02100010????1110???1?2?1?110211111111?????1?000010100000???1?0??0110112111011111??1111?110101101?00101021201110???1??1????0?????0000?0?

Nomingia_gobiensis

??????????????????????????????????????????????????????????????????????????????????????????????????????00?1?01?00101020????????????????111011011111011111111??????????????????????1?00????????????????????111???0???1???1????????1??10????????????????????????

Epichirostenotes_curriei

?????????01?010??????1????????????????????????????110?1?1??0???????????????????????????????????????????2?1?111??1???2????????????????????????????021?????????????????????????????1??????????????????????111??0?0???1???????????????10??0?????????????????????

Banji_long

11?210111???0111102121111111011??11101????0??11?1111????????1111121?0111??11?2121??000110??1?11?122?1???????????????????????????????????????????????????????????11111001011??????????110??????0?00000????????????????00?0?000?0???????0??????????????????????

Caudipteryx_dongi

?????????????????????????????????????????????????????????????????????????????????????????????????????????01?0?????????????0010?1?20100?011?2011000211?????100012????????????00?031000?????????????????01?10?????0??1?????????????00000???????000000000????110

Ganzhousaurus_nankangensis

???????????????????????????????????????????????????????????????????????11111?21?2011001?????111???2????????????0???0???????????????????????????????????????0001????1?001?11???????1????????0?001?10?0???????0???????????111???0??????????????000000000?????0?

Jiangxisaurus_ganzhouensis

1???????????????????????11???????1?001????????11????????????1??1??????111111??101?1?0011?111????122??10?????????1???????1?1100?0?10????0???????????????????????????1?001?10??2????????????????0?00000?0?????????1?0??????????00??00???????????????????????001

Nankangia_jiangxiensis

???????????????????????????????????????????????????????????????????????11101?2?????100????????????2??????1???1??1??0?0011001?????????0111011111111211001111????????1101??0???????1?00??11?????0?1???1????11???????01???11?0???0??0?00????????????????????????

Shixinggia_oblita

?????????????????????????????????????????????????????????????????????????????????????????????????????????1?2?1??1?????????????????????110000000?????1????1?????????????????????????01????????????????????????????????????????????????????????????????????????

Similicaudipteryx_yixianensis

??????????????????????????????????????????????????????????????????????????????????????????????????????0?11?0?1?0??1?2???????1????????0?001?21110?????????0??0?12???????????????????00???????????????????????0???????????????????10?????0?????000?0000????????

Wulatelong_gobiensis

11?2211?11?1?1???02121?111?1??1?111001??00??11???????????????1111???????????????101????1??11????12???????1???100????2??????????1??????1100?211111121???????000?01???????????0??????011????????????000????1????1?10?101????????????0??0??0???????000000????0??

Yulong_mini

1020101110110110111121010111010?01000111010?111110110??0????????0?????11111112122011001111111?12122?100??1????001?10??1??0000101?21010?110?10??0??0?1?0??11?001010?11001011?11000?100110??????0?0000010???????1??????1110?01000??00???00?????000000000????10?

Protarchaeopteryx_robusta

?????0????????????????????????????????????????????????????????????????????0????????0????????????0?0??????1????????1?????????1011?21111?100?011?0???????????000120???????????00003?000??0??????????????00????????0????????????????00??0?0?????000000000????100

Xingtianosaurus_ganqi

??????????????????????????????????????????????????????????????????????????????????????????????????????????????00011??00???0011?1021010???122????0??1???10??00?12???????????000??010???????????????????0??10?0???1?01????????????000000????11?0??0???????0?100

4.3 *Xingtianosaurus ganqi* in another phylogenetic analysis

We added *Xingtianosaurus ganqi* in the phylogenetic analysis of Brusatte et al. (2014). The following are the character state scores of *X. ganqi*

????????????????????????????????????????????????????????????????????????????????????????????????????????????????1?10?0300???001?????????11?00??00000001?0??????000201202?1?10??0?00????0????00????00000?01000?0??00?????????????00?0010??????????????????????????????200????????????????????????????????????????????????0?0??0?00?0???????????????????????10??11????0?0000??00?00???????1????1?1010?0??000??0????????000?????1??1???00????0000??00?0000000????????01??????????????????0?000?????????????????????????????????????????????????????????????????????????????????????????????????????????????????????????????????????????????????????????????????????????????????100????????????????????????????1???000000???????????????????????0???0???????00???????100?00??1????????????????0??00????????????????????????0??????00?????0?????1??????0??00?0???????0??0?????????????????
